# A Study Protocol to Determine Heat-Related Health Impacts among Primary Schoolchildren in South Africa

**DOI:** 10.3390/ijerph17155531

**Published:** 2020-07-31

**Authors:** Shalin Bidassey-Manilal, Caradee Yael Wright, Thandi Kapwata, Joyce Shirinde

**Affiliations:** 1 Environmental Health Department, Faculty of Health Sciences, University of Johannesburg, Johannesburg 2028, South Africa; Caradee.Wright@mrc.ac.za (C.Y.W.); Thandi.Kapwata@mrc.ac.za (T.K.); 2 Environment & Health Research Unit, South African Medical Research Council, P.O. Box 87373, Houghton, Johannesburg 2041, South Africa; 3 The School of Health Systems and Public Health, Faculty of Health Sciences, University of Pretoria, Private Bag X323, Arcadia 0007, South Africa; joyce.shirinde@up.ac.za

**Keywords:** climate change, high temperatures, primary schoolchildren, heat-related health symptoms

## Abstract

Climate models predict that the global average temperature of Earth will rise in the future. Studies show that high classroom temperatures can affect the ability of the student to learn and function. It is important to understand the impact that heat will have on the health, wellbeing, and academic performance of learners, as they spend a significant amount of time in classrooms compared to any other environment. A follow-up panel study among 20 public primary schools in the Gauteng province (South Africa) will be carried out, in which Grade 4 learners will be selected to complete an hourly heat-health symptom questionnaire. A Cambridge Neuropsychological Test Automated Battery (CANTAB) test will be used to determine their memory and attention span. A nursing practitioner will measure body weight, height, and temperature. Lascar data loggers will be used to measure indoor classroom temperature. School principals will complete a questionnaire on existing school coping mechanisms and policies in place that help deal with hot weather conditions. This is the first study to quantitatively assess the effects of heat on learners’ health, well-being and school performance in South Africa. The outcomes of this study will enable policymakers and public officials to develop appropriate school heat adaptation and mitigation measures and will assist in channeling their resources where it is most needed.

## 1. Introduction

Many high-income countries have addressed the issue of vulnerability to heat and its associated health impacts among schoolchildren; however, limited data are available for low- and middle-income countries [1]. Previous studies predicted that temperature across the African continent, which will likely be most affected by the effects of climate change, will rise by 2 to 6 °C over the next 100 years [2,3]. Model projections of change in future mid-summer temperature patterns over southern Africa project an increase of more than 4 °C, approximately twice the global rate of temperature increase [4].

There is currently an absence of guidelines to specifically deal with indoor thermal comfort in school classrooms. A review of the literature shows that researchers, scientists, architects, and engineers are currently using existing occupational building standards to compare it to educational settings [5,6,7,8]. A review of literature on thermal comfort inside classrooms revealed that there are studies available on thermal comfort in educational settings; however, the majority of these studies have been carried out at tertiary institutions [9]. There are few studies that have been administered at secondary schools and literature available on thermal comfort in primary schools indicate that most of them have been carried out to determine thermal comfort during winter. Over the past few years, there have been several studies conducted throughout the world on the effect of heat on schoolchildren while at school; however, limited studies of this nature have been carried out in South Africa [9,10].

Two field experiments were carried out in classrooms in Denmark by Wargocki and Wyon [11] during late summer among 10 to 12-year olds (2004–2005). In the first classroom, temperature was reduced or cooled from 25 °C to 20 °C and ventilation altered increasing outdoor air supply rate. In the second classroom, the air temperature was changed but the outdoor air supply rate remained constant. The learner performance of two numerical and two language-based tests improved significantly when the temperature was reduced, and increasing the outdoor air supply rate improved the performance of schoolwork by the children [11].

A study was conducted in Cameroon in order to evaluate the impact of heat on learners aged between 12 and 16 years in public schools. Adverse symptoms such as headache, fatigue, and feeling very hot were found to be associated with high indoor air classroom temperatures. Poor school performance was also observed during the warmest period of the day [12].

In 2016, a largely qualitative study to determine the perceived health impacts of heat on learners was conducted in Johannesburg, South Africa. Two hundred and fifty learners aged between 14 and 18 years from eight high schools completed an hourly heat-health symptom log over five days. Data loggers measured indoor classroom temperatures that coincided with a learner symptom questionnaire. The results showed that a high proportion of students felt tired (97%), had low levels of concentration (96%), and felt sleepy (94%) during at least one hour on any day. There were statistically significant correlations when controlling for school cluster effect and time of day, between indoor temperatures > 32 °C and students who felt tired and found it hard to breathe [13].

The government of South Africa has been generating strategies, policies, and plans that respond to a growing awareness of the impacts of climate change [14]. For example, the South African Climate Change and Health Adaptation Plan 2016–2019, and The Integrated School Health Policy and The National Climate Change Response Strategy [15,16]. Soon, a National Heat Health Action Plan for South Africa will be published, and this includes guidance for addressing heat in schools [17]. The implementation of these policies and plans that help to address heat and children in schools are needed. Several schools around the country are overcrowded, most are not fitted with air conditioners and rely on natural ventilation, whilst others have no water or proper infrastructure, a situation that may result in poor thermal comfort in the classroom [18]. This is likely to have negative impacts of perceived health of learners. Therefore, the aim of this study is to describe the effect of heat in the classroom on primary schoolchildren’s thermal comfort and cognitive function in government primary schools in South Africa. The objectives include quantification of indoor and outdoor temperature and humidity; understanding schoolchildren’s perceived hourly heat-health symptoms using a self-reported questionnaire, and daily lifestyle practices using a nurse-administered questionnaire; conducting body screening and cognitive function tests for verification of schoolchildren’s perceived heat-related symptoms; and, assessing school infrastructure and school policies in relation to heat and health guidelines. These findings will be used to inform recommendations that can be used for the development of holistic guidelines for South African primary schools on how to manage hot weather conditions in relation to classroom thermal comfort. Given the exponential growth in studies assessing climate-related impacts on health, it is prudent to share study protocols in advance to solicit feedback and identify best practices for such studies. Hence, the presentation of this study protocol—prior to pilot testing—that aims to determine the effect of heat in the classroom on primary schoolchildren’s thermal comfort and cognitive function. We hypothesize that high temperatures in classrooms have an effect on the learning ability and cognitive function of schoolchildren. We also hypothesize that assessing indoor thermal comfort levels and perceived heat health symptoms that are based on exposure to elevated apparent temperature would help us to understand better schoolchildren’s susceptibility to heat stress.

## 2. Materials and Methods

### 2.1. Study Setting and Sample

**Figure 1:**
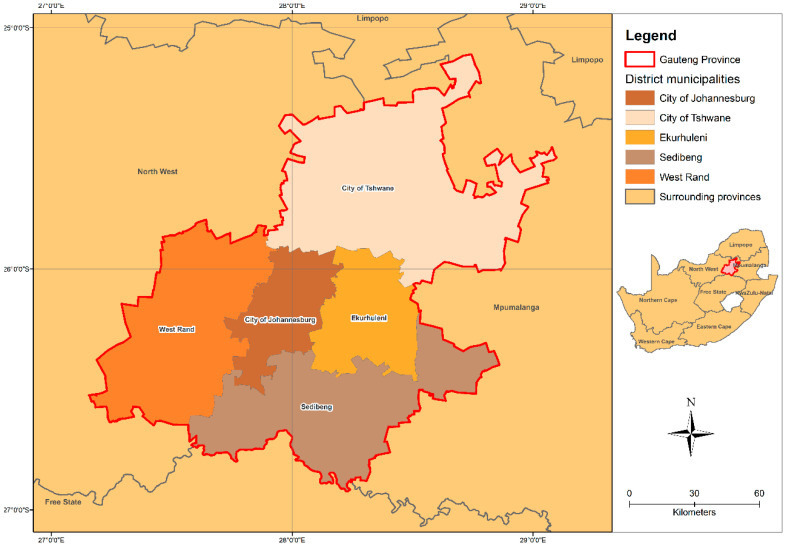
Map of Gauteng Province and its five municipalities (Source: Thandi Kapwata, South African Medical Research Council).

The researchers designed a follow-up panel study to determine the effect of heat in the classroom on primary schoolchildren’s thermal comfort and memory and attention span in South Africa. This study will take place in 20 public primary schools in the Gauteng Province of South Africa (Figure 1). Gauteng consists of five municipalities, namely Johannesburg, Tshwane, and Ekurhuleni, and two smaller districts, Sedibeng and West Rand [19]. With an area of 18,182 km^2^, Gauteng is the smallest province in South Africa, but is densely populated (approximately 15.2 million) [20]. In South Africa, public schools are classified into five categories based on the relative poverty levels of the geographic area from which schoolchildren are eligible to attend a particular school. Quintile 1–3 are ‘no-fee’ schools that are mainly dependent on financial support from the Department of Education; quintile 4–5 refers to schools where parents pays fees for the cost of education and these schools tend to have few infrastructure-related backlogs. More than 47% of Gauteng schools fall within quintile 1–3 [21]. There are currently more than 2000 public primary schools in the province and this study sample will include schools from all five quintile categories. Temperatures over the central and inner parts of Southern Africa including the Gauteng province are projected to increase between 1 °C to 2 °C in the near future accompanied by very hot days and heatwave events [22]. Therefore, it is crucial to understand heat-related health impacts among schoolchildren in public schools, as most of them have limited resources and are not equipped to deal with hot weather conditions

Learners in Grade 4 (class size usually ranges between 30–40 children), including both males and females from government primary schools, will be selected to participate (inclusion criteria) in this study. Learners whose parents do not give consent to participate in this study will be excluded (exclusion criteria) from the study. The researcher will request a list of public primary schools from the Gauteng Department of Education. Thereafter, a clustering method will be used to identify 20 schools in the province from which schools with Grade 4 learners will be randomly selected to participate in the study. Once the list of learners is received from the school, the names will be captured on a Microsoft Excel spreadsheet and randomly selected. This method of selection eliminates bias and gives all of the participants an equal opportunity to participate. Principals of the 20 schools will also participate in this study by completing a questionnaire regarding school policies, procedures, and coping mechanisms that are available to deal with hot weather conditions.

### 2.2. Data Collection

This study adopts both qualitative and quantitative data collection techniques. The study will take place over two days (20 schools × 2 days = 40 days) in each school and over summer months (i.e., in February and March 2021) when temperatures are warm in Gauteng. Data will be collected in different phases using seven different tools.

**Figure 2:**
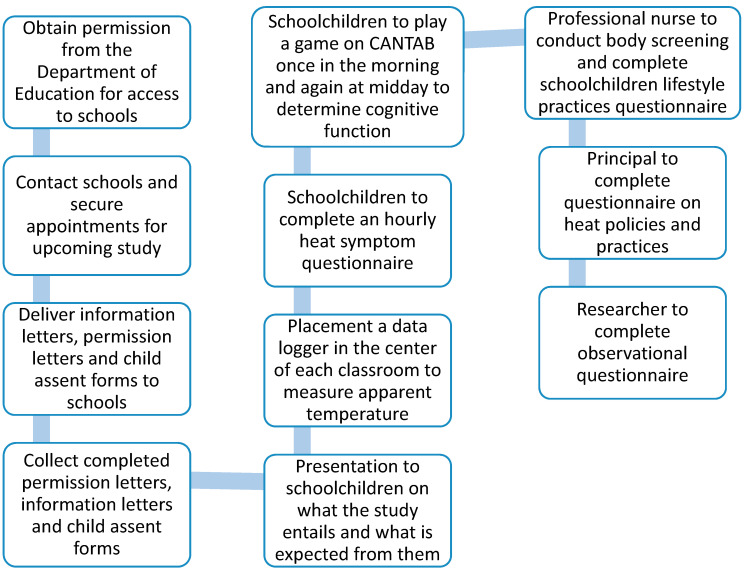
Study flowchart.

Figure 2, below, represents the study flowchart. Firstly, the team will request permission from the Gauteng Department of Education to gain access to public schools. Secondly, the team will approach the parents or legal guardians of learners for consent to include their child in this study as well as the school governing body and principals. Lastly, after receiving parental consent, the team will approach all of the learners from the schools for assent to participate in the study prior to commencement. A temperature logger will be placed in the centre of each classroom, schoolchildren will then play a game on CANTAB and fill in a heat-related symptom questionnaire and a nursing practitioner will be available for screening and completion of a lifestyle practices questionnaire. School principals and the researcher will also be required to complete questionnaires.

The seven different tools that will be used for this study are as follows:

Tool 1—Data Logger:

Heat stress index measured as apparent temperature (temperature and relative humidity herein referred to as temperature) will be used to assess thermal comfort in the classroom. A data logger (an instrument that measures temperature and relative humidity) will be installed in each classroom and it will be used to measure data at 10-min. intervals and store data relating to indoor temperature, dew point, and humidity. The logger will be placed in the centre of the classroom and secured with a length of string. The measurement range of the loggers is between 0 to 30 volts direct current, they have a resolution capability of 50 medium voltage direct current, and are able to conduct readings per cycle 32 510 with a user selectable between 1 s and 12 h and a battery life of three years [23]. Data will be downloaded to a computer and then graphed or exported to other applications. All equipment will be calibrated and maintained according to manufacturer specifications.

Temperature and relatively humidity will be used to calculate apparent temperature (AT). AT is an adjustment to the ambient temperature that is based on the level of relative humidity and it is deemed a measure of how humans perceive or feel temperature. AT is a frequently used indicator of probable human physical reaction to weather conditions and several studies have used AT in order to examine the association between health and high temperature [24,25,26,27]. Hourly AT will be calculated using the following equations:

AT = Ta + 0.33 × e − 0.70 × ws − 4.00(1)

where Ta = dry bulb temperature (°C), e = water vapour pressure (hPa), ws = wind speed (m/s) at an elevation of 10 m (set to 0 because this was an indoor setting)

e = rh/100 × 6.105 × exp (17.27 × Ta/(237.7 + Ta)(2)

where rh = relative humidity (%).

Currently, no comprehensive relationships between AT or temperature ranges and their effect on human health are available for Africa [28]. Therefore, an international symptom table developed by the United States National Weather Service (USNWS) and the National Oceanic and Atmospheric Administration (NOAA) will be used in this study. These temperature thresholds and likely health effects have been used in similar studies in the African context [29]. The calculated indoor ATs were compared to USNWS NOAA heat health effects, as shown in Table 1 [30], to estimate potential temperature ranges to which learners may be exposed and consider the temperature-associated health risks.

We will also calculate thermal comfort according to ASHRAE Standard 55–2017 using a web-based tool created by the University of California at Berkeley [31] for comparison with AT. This method includes six environmental and personal factors: temperature (from the data loggers), mean radiant temperature (obtained from a globe thermometer), humidity (data logger), air speed (indoor therefore set to 0), activity level (metabolic rate), and occupant clothing (degree of insulation). These six factors will be used to determine whether thermal comfort in classrooms is within the required limits using a psychometric chart with superimposed comfort zones for summer and winter conditions [32].

Tool 2—Anthropometric and Body Temperature Measurements:

A nursing practitioner (recruited through an agency of nurses and registered with the Health Professions Council of South Africa (HPCSA) will be recruited to measure height, weight, and temperature using a digital measuring tool (for height), digital weighing scale (weight), and a digital thermometer (temperature). Measurements of weight and height will be done once during the study and temperature measurements will be taken on a chosen day (morning from 08:30–10:00 and after midday from 12:30–14:00). All of the screenings will be conducted in a private space. The results obtained from the measurements will be used to determine whether there is a relationship or correlation between learner anthropometrics, high classroom temperature, and cognitive function.

Tool 3—Nurse Questionnaire:

Nurses will complete a questionnaire/form with the above measurements and six questions regarding learners’ thermal comfort, indoor temperature preference, their diet plan for breakfast and lunch, and their daily water consumption levels.

Tool 4—Learner Symptom Questionnaire:

A questionnaire designed for learners will be self-completed by learners in order to obtain information on their background (age, grade) and health symptoms (relating to body heat/discomfort) experienced at schools specifically during warm days. There are eight questions per questionnaire that learners will complete hourly. The results obtained from the learner questionnaire will coincide with the results from the data loggers. Table 2 provides the eight questions.

Tool 5—CANTAB:

The Cambridge Neuropsychological Test Automated Battery (CANTAB) (developed by the University of Cambridge) is a computer-based cognitive assessment system that consists of a battery of neuropsychological tests administered to participants while using a touch screen computer or tablet. This cognitive software will be used to determine reaction time, attention span and information processing ability of learners. This software is provided on a tablet device that learners will use to play a game and the results obtained from the game can help the researcher to evaluate the performance of learners at that point in time.

The test will be carried out in the morning, and again at midday when ambient temperatures are higher than temperatures during the morning. Learners will be randomly selected to conduct these tests. Each test should take at least 2 min. to complete. Studies using this software have been carried out by the research team in previous studies. All of the researchers in this study will be trained on the use of this software prior to the commencement of the study.

Tool 6—Observational Questionnaire:

The researcher will complete an observational questionnaire to record the topographical location of the school, the hot weather practices in the classroom, the school infrastructure (building materials used to construct the school, ventilation and lighting), and the effects that the infrastructure may have on the thermal comfort of learners in the classroom. The results obtained from the observational questionnaire will be used to determine whether there is an association between the observational variables and the classroom temperature recorded. The School Infrastructure Safety and Security Guidelines (SISSG) and the regulations relating to minimum uniform norms and standards for public school infrastructure of South Africa were developed to guide the planners, designers, decision-makers, and the sector at large on how to incorporate safety and security measures when providing school facilities [33,34].

Tool 7—School Principal Questionnaire:

A questionnaire will be administered to school principals on school coping mechanisms implemented for hot weather conditions. This questionnaire will help the researcher to gather information on what guidelines or policies are available (or not) at schools when temperatures are hot and, should a heat-related emergency arise, what steps would be implemented to ensure learner and teacher safety. The questionnaire will also help the researcher to gather information on the type of uniforms (as clothing is a factor that affects the thermal comfort of a person) that are used in schools and the time slot allocated to physical education during school hours (i.e., during hot times of the day). Our findings will be considered against existing protocols and guidelines, such as The Ottawa Carleton District School Board Extreme Weather Conditions School Protocol, County of Orange California hot weather guidelines, etc. [35,36]

Using outcomes from tools (1) to (7), recommendations will be made that can be used for the development of guidelines for South African primary schools on how best to manage school arrangements and protect children’s health during hot weather.

### 2.3. Quality Control

All of the questions and demographic information will be coded, so it will not be linked to the researcher’s name. All personal information regarding research participants will be collected, stored, and once used destroyed in ways that respect the privacy and confidentiality of the participants. All of the information obtained from the field will be kept in a locked storeroom in the researcher’s office. The information obtained will be utilised for research purposes only.

Electronic data will be stored on a laptop that is only accessible to the research team. The laptop and the data file will be password protected. All records of the participants will be kept for a period of five years and then thereafter destroyed. Double entry of data will be done in order to prevent any misinterpretation or errors. All received responses will be coded prior to capturing for statistical purposes.

In terms of the instrumentation, the loggers that will be used for temperature monitoring are supplied with a calibration certificate as well as software configuration. Throughout the research, validity and reliability will be ensured by applying recognised and acceptable statistical procedures and methods. The researcher will further ensure the questionnaire and interviews are pre-tested for reliability and validity.

All of the instruments used in this study will be calibrated according to the manufacturer’s specifications prior to the commencement of this study. Assistance will be sought from an expert in the field for guidance during data collection and analysis. Body temperature, weight, and height measurements will be carried out by a professional nurse, currently registered with the Health Professions Council of South Africa (HPCSA) or/and South African Nursing Council (SANC).

### 2.4. Data Management and Analysis

Questionnaire data will be captured into Microsoft Excel. Quality control measures to maintain data integrity will include finding and rectifying inaccurate or corrupt data and removing duplicates prior to analysis. Data will be analysed using R Studio version 3.6, as this is a multi-level study with multiple types of data collection tools that will be used [37].

Descriptive statistics will be used to summarize all variables of interest. The Pearson chi-square test and an independent *t*-test will be used to determine differences in participant characteristics. Boxplots will be used to show the prevalence of heat related health symptoms experienced by learners at hourly intervals from 8:00 am to 3:00 pm. These symptoms include fatigue, dizziness, loss of concentration, thirst, and generally feeling unwell. Based on our previous study [13], it is expected that the largest number of heat related symptoms will be experienced after 12:00 pm when indoor temperatures peak.

Generalized additive mixed models will be used to estimate the effects of indoor apparent temperature on cognitive function. Models will be adjusted for school quintile, building material, and participant age, sex, and BMI. We expect to observe a decrease in cognitive function as apparent temperature increases, the threshold at which this decrease begins to occur will be obtained from the results.

Multiple logistic regression analysis will be used to determine the relationship between heat related symptoms reported by learners and apparent temperature. The significance of these results will be examined using *p*-values, ORs, their corresponding 95% Cis, and the overall model Rho. Models will again be adjusted for school quintile, building material, and participant age, sex, and BMI. We hypothesize that there will be a higher risk of school children experiencing heat related symptoms as indoor apparent temperature increases. Statistical significance will be assigned at *p* < 0.05 for all analysis.

Student differences in perceived heat-related health symptoms by non-modifiable and modifiable risk factors will be explored using Random Effects (RE) panel data logistic regression models, all models controlled for school. The significance of these relationships will be explored using *p*-values, odds ratios (OR), their corresponding 95% confidence intervals (CI), and the overall model Rho. RE models will be used so that the time invariant variables (such as sex) remain in the models.

Temperature and humidity data will be plotted using time series analysis for each school by day of the week. Interaction analyses using repeated measures analysis of variance (ANOVA) will be used to check whether the effect of temperature on cognition, as assessed by the composite score (average test score from the CANTAB games), is modified by as age, gender, BMI, body screening results, or school. For interaction analyses, interval variables will be stratified into two levels while using the median value. Classroom indoor apparent temperature will also be compared to the USNWS NOAA Heat Index symptom bands and ASHRAE standard for thermal comfort to establish the thermal comfort levels experienced by children.

### 2.5. Ethical Procedure

A submission for ethics clearance for the research was submitted on 12 June 2019. Ethical clearance (REC-01-34-2019) was granted by the Research Ethics Committee of the University of Johannesburg, and this protocol was approved by the Higher Degree Committee (HDC-01-29-2009). Permission to work in the province was received from the Gauteng Department of Education [1,2,4,8]; however, to date, no fieldwork has begun.

Prior to participating in the study, all of the participants will receive a detailed information leaflet and consent forms from the University of Johannesburg. All documents will be available with an explanation of the aims, objectives, and duration of the research and participation prior to giving consent. The study will be carried out with parental consent for minors as well as consent from the principal and school governing body before the commencement of the study.

Participant autonomy and confidentiality will be respected; participants will be managed as unique human beings and their freedom of choice safeguarded. Consent will be voluntary, without duress, coercion, or bribery. The participants may withdraw from the study at any time without consequence. No harm is anticipated to come forth from participation in this study. The collected data will be used for research purposes only. Privacy will be guaranteed due to the sensitivity of the information provided and the questions posed. Information given by the participants or discovered during document audit will be treated as confidential. The researcher will also ensure that participants complete the questionnaire without intrusion. No acts will be performed that could humiliate the participants. A trained nursing practitioner will conduct all screening for learners in private to prevent embarrassment and bullying.

One of the main goals of this study is to provide recommendations for schools on how hot weather conditions can be managed effectively. We aim to disseminate the results from the study to the NDoE (National Department of Basic Education), schools that will participate in the study, and policymakers in the health sector through presentations and an official report. The results obtained from this study will be presented at accredited scientific conferences and published in a peer-reviewed journal.

## 3. Discussion

Studies have shown that rising temperatures influence student health and performance [11,38,39]. School marks an important early milestone in the child’s lifelong journey of intellectual and psychosocial development [39]. Therefore, an understanding of the child’s environment should have prominence in design considerations for a child-friendly school. This will help to reinforce the child’s self-identity, promote a sense of belonging to a place, and group [40].

Classrooms are functionally quite different when compared to other built environments, such as offices and residential dwellings, as they are learning environments and safe spaces conducive to learning. However, in terms of thermal comfort no existing standards or guidelines exist besides those for occupational settings, which have been used as comparison to educational settings [5,40]. Thermal preference and comfort studies on adults cannot be extrapolated to children because of differences in developmental characteristics that may lead to different responses in adults and children [9,41]. Children are one of the high risk groups of a warming world, as they are more susceptible to the effects of exposure to high temperatures, but they are also the most powerful protagonists for change [41]. The majority of government schools in Gauteng are not equipped with artificial ventilation and cooling systems and, thus, rely on natural ventilation for thermal comfort. There are a few schools that have ceiling fans to help with high ambient temperatures, but considering that most schools are overcrowded and have small classrooms the effectiveness of a ceiling fan becomes questionable [13]. This study will provide evidence of the effect of apparent temperature on children’s learning ability.

## 4. Conclusions

Gaps still exist in the knowledge of heat-related health impacts among schoolchildren and in the context of climate change and predicted rising temperatures, the urgency to fill these gaps is pressing. The next steps in the proposed study will include: piloting of the study, securing funding, appointment, and training of two support staff and a professional nurse, setting up appointments with schools and school governing bodies (study has been approved by the Department of Education) for permission to carry out the study at a randomly selected school, handing out information letters, consent and permission letter to learners for parents’ permission to participate in this study, data collection, data analysis, and write up. This study will contribute to the knowledge gaps on the effects of hot weather conditions on primary schoolchildren in South Africa, Africa and other low- and middle-income countries.

## Figures and Tables

**Table 1:** Apparent temperature ranges and associated possible heat health effects according to the USNWS NOAA Heat Index.

Level	Apparent Temperature Range	Warning	Possible Heat Health Effect
1	26.7–31.7 °C	Caution	Fatigue, discomfort possible
2	32.2–40 °C	Extreme caution	Sunstroke, heat cramps, heat exhaustion possible
3	40.6–53.9 °C	Danger	Sunstroke, heat cramps, heat exhaustion likely, heat stroke possible
4	54.5 °C or higher	Extreme danger	Sunstroke and heat stroke highly likely

**Table 2:** An example of a completed Learner Symptom Questionnaire of how it will be completed daily.

Number	Questions	Hours						
		Date: DD MM YYYY						
		08:00–08:59	9:00–9:59	10:00–10:59	11:00–11:59	12:00–12:59	13:00–13:59	14:00–15:00
1	Was there a time during the day when you felt tired?		×					
2	Was there ever a time during the day that you had a headache?						×	
3	Was there any time during the day you found it hard to pay attention in class			×				
4	Was there any time during the day that you felt sick?						×	
5	Was there any time during the day that you felt dizzy?					×		
6	Was there any time during school hours that you had nausea or felt like throwing up?						×	
7	Was there any time during the day that you felt thirsty?		×					
8	Was there a time during the day when you felt sleepy or slept in class?					×		

## Abbreviations

HPCSA	Health Professions Council of South Africa
NDoE	National Department of Basic Education
NDoH	National Department of Health
USNWS	United States National Weather Services
NOAA	National Oceanic and Atmospheric Administration
CANTAB	Cambridge Neuropsychological Test Automated Battery
SANC	South African Nursing Council
STATA	Software for Statistics and Data Science
RE	Random Effects
OR	Odds Ratio
CI	Confidence Intervals
